# Different coupling mechanisms for a novel modular plate in acetabular fractures—a comparison using a laparoscopic model

**DOI:** 10.3389/fsurg.2024.1357581

**Published:** 2024-06-11

**Authors:** Maximilian M. Menger, Steven C. Herath, Andreas E. Ellmerer, Alexander Trulson, Max Hoßfeld, Artur Leis, Annika Ollig, Tina Histing, Markus A. Küper, Christof K. Audretsch

**Affiliations:** ^1^ Department of Trauma and Reconstructive Surgery, BG Trauma Center Tuebingen, Eberhard Karls University Tuebingen, Tuebingen, Germany; ^2^ Department for Orthopaedics and Traumatology, Medical University of Innsbruck, Innsbruck, Austria; ^3^ Department for Traumatology, Orthopedics and Surgery, BG Trauma Center, Murnau am Staffelsee, Germany; ^4^ Institut für Strahlwerkzeuge (IFSW), University of Stuttgart, Stuttgart, Germany

**Keywords:** acetabulum, laparoscopic, plate, fracture, osteosynthesis, endoscopic, minimal-invasive

## Abstract

**Introduction:**

Acetabular fractures are among the most challenging injuries in traumatology. The complex anatomy usually requires extensive surgical approaches baring the risk for iatrogenic damage to surrounding neurovascular structures. As a viable alternative, minimally invasive endoscopic techniques have emerged during the recent years. This paper reports on the feasibility of different coupling mechanisms for a novel suprapectineal plate especially designed for minimally invasive acetabular surgery.

**Methods:**

A total number of 34 participants contributed to the present study, who differed in their arthroscopic and surgical experience. A laparoscopic model was used to compare four different coupling mechanisms by the number of failed attempts, the time required for plate fixation, the influence of surgical experience as well as the learning success for each individual coupling mechanism. Moreover, the feasibility of each mechanism was evaluated by a questionnaire.

**Results:**

The results demonstrate that plates employing grooved and pressure-sliding coupling mechanisms exhibit fewer failed attempts and reduce trial times, especially in contrast to sole sliding mechanisms. Furthermore, our study revealed that proficiency in endoscopic procedures significantly influenced the outcome. Notably, the subjective evaluation of the participants show that the pressure base and pressure-slide base plate designs are the most supportive and feasible designs.

**Conclusions:**

In summary, the present study evaluates for the first-time different plate and coupling designs for minimal-invasive surgery, indicating a superior feasibility for plates with a grooved and pressure-sliding mechanism.

## Introduction

Acetabular fractures are among the most demanding injuries treated by trauma surgeons (1). The complex three-dimensional anatomy of the acetabulum, the proximity to vital anatomical structures such as iliac vessels, obturator neurovascular bundle or the urinary bladder pose a major challenge for the treating surgeon (2). Due to this complex anatomy, extensive surgical approaches such as the posterior Kocher-Langenbeck approach or the anterior ilioinguinal approach are usually necessary to achieve an open reduction and adequate fixation of the acetabular fracture (3, 4). Notably, the associated risk of iatrogenic injuries to neurovascular structures should not be disregarded.

Recent reports demonstrated in both, cadaver studies and single case reports, the complete endoscopic preparation of the pelvic ring and acetabulum, which is based on laparoscopic pelvic lymphadenectomy, a procedure mostly performed in urologic and oncological surgery (5). Moreover, even endoscopic plate osteosynthesis of the pelvic ring or acetabulum are described in literature (5, 6). However, using these minimally invasive endoscopic procedures can be highly demanding and a feasible implant technology is of major importance for ensuring a successful osteosynthesis. Thus, the plate and coupling mechanism should be easy to handle and the laparoscopic insertion of the plates should be easy to learn even for surgeons with less experience in camera-assisted surgical techniques. Moreover, due to the lack of endoscopic procedures in trauma surgery, appropriate implant technologies are missing, especially for the adequate plate alignment and fixation. Therefore, we herein investigated in the present study the feasibility of different coupling mechanisms for a recently developed modular suprapectinal plate with a buttress plate for the quadrilateral surface (7). The aim is to identify the coupling mechanism with the highest overall success rate. Coupling mechanisms offering a wide range of movement possibilities, such as linearly displacement before tightening and tilting of the plate in a variety of angles, should provide the most intuitive handling and subjective feasibility.

For evaluation of the usability of the different types of modular plates, we used a laparoscopic model and analyzed the number of failed attempts for plate fixation and the time required for successful plate fixation. Moreover, the influence of surgical experience and the learning success for each individual coupling mechanism was assessed.

## Materials and methods

### Types of coupling mechanisms

**Figure 1:**
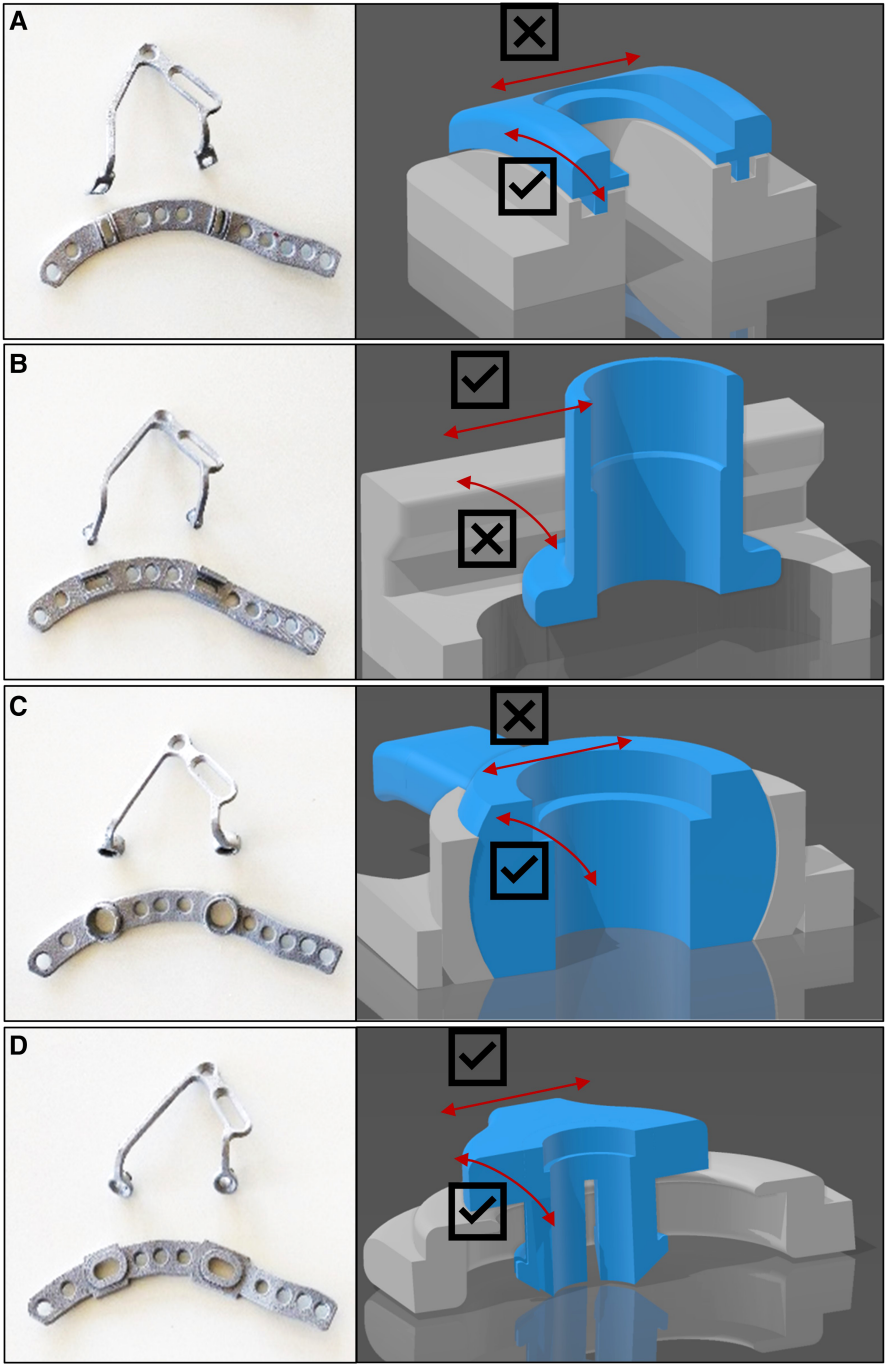
The four coupling mechanisms: Four mechanisms for mechanical coupling of the plate elements. (**A**) The grooved plate (plate 1) with sliding grooves to which the plate can be attached. (**B**) The sliding plate (plate 2), which has two ends that must be threaded into a rail system. (**C**) The pressure plate (plate 3) inserts via its two ends like pushbuttons. (**D**) The pressure-sliding plate (plate 4) is pressed into the fixed plate in a similar way to the pressure plate but can still be moved in an oval window afterwards.

Each of the four plate designs considered in the handling study consists of two separated parts and was: First, a base plate anatomically shaped along the pelvic brim and designed for suprapectineal placement. Second, a buttress plate segment, which provides the support for the quadrilateral surface. Base and buttress plate can be coupled by the respective coupling mechanism examined in this study. The buttress plate segment has a V-shaped design and matching coupling elements for the base plate on both ends. The base plate segment as well as the buttress plate segment are configured in the same way in all four variants except for the coupling mechanism and, when the plates are coupled together, always result in the same three-dimensional structure (see Figure 1 for an example).

The grooved base plate (plate 1) incorporates sliding grooves into which the buttress plate can be hooked using the tongues provided. The tongues engage in the grooves, ensuring correct orientation to each other, especially during bolting. In addition, after coupling and before tightening, the buttress plate can be displaced from ventral to dorsal, orthogonal to the linea terminalis, as with a friction bearing, and thus tilted at a defined angle (Figure 1A).

The sliding base plate design (plate 2) incorporates two ends to be placed and moved within a railing system, which then can be bolted together. This offers the user the possibility to flexibly adjust the placement of the drill holes in a range of approximately 6 ± 0.5 mm after insertion. The module buttress plate must be threaded in at the correct angle considering the position of the both ends at the same time, as the V-shaped buttress plate is not deformable in itself. From this, it cannot be bent apart to fit into a misaligned base plate. It can be moved medially-laterally in the railing system in the direction of the linea terminalis, but the connection cannot be tilted along the moving axis (Figure 1B).

The pressure base plate (plate 3) is characterized by the coupling mechanism via its two ends similar to a “push button”. Due to this design it is fixed in a defined position and remains after being inserted without additional bolting. The buttress plate can easily be detached from the base plate by a small pull. Since the position of the hole is not adjustable afterwards, its correct position has to be defined already when positioning and fixing the base plate. Due to the geometrical shaped coupling mechanism the buttress plate can also be tilted within the predefined position (Figure 1C).

The pressure-slide base plate (plate 4) shows a combination of the coupling mechanisms of the sliding base plate and the pressure base plate. Here, the buttress plate is pressed into the already fixed base plate in a similar way to the pressure plate but can still be moved linearly after insertion, similar to the sliding base plate. The buttress plate remains coupled with the base plate after being pressed inside without additional bolting, but the hole for fixing the buttress plate can still be flexibly adjusted in a range of 3 mm ± 0.5 mm. Furthermore, this module is easily adjustable in angle (Figure 1D).

### Participants

Over a period of 3-weeks, we enrolled 34 volunteers at the BG Klinik Tübingen for our study. This group consisted of diverse medical backgrounds: one non-medical individual, four physician assistants, ten medical students, 16 residents, two specialists, and one senior physician, all specialized in trauma surgery. Within this cohort, 24 participants had no prior exposure to arthroscopy, seven had up to one year of experience, and three had one to three years of experience. As for laparoscopy, 28 participants were newcomers, while three had less than a year of experience, one had one to three years of experience, another had three to five years, and one participant had more than five years of laparoscopy experience.

### Experimental design

**Figure 2:**
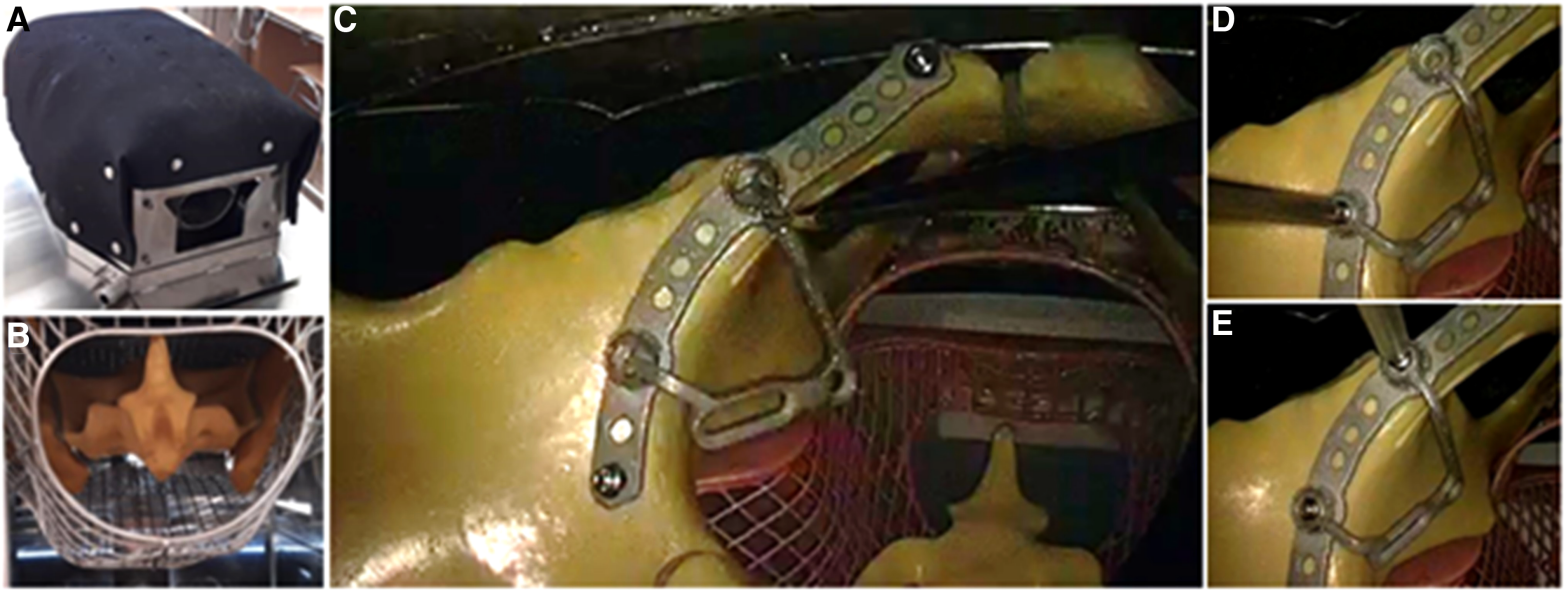
Experimental setup: (**A**) The laparoscopic training torso in which the SYNBONE pelvis is located. (**B**) View from below into the training torso, the pelvis with sacrum can be seen. (**C**) View via endoscope from cranial into the pelvis lying in the training torso. The buttress part of the plate is being connected to the baseplate part that has already been inserted and is fixed (**D**) with a dorsal and (**E**) ventral screw.

To test the coupling mechanism, the four different plate models for the left acetabulum were fabricated from aluminum using 3D printing (Figure 2). Four pelvis models (Full pelvis, SYNBONE AG, Zizers, Switzerland) were prepared with the four different base plates, which differed in their coupling mechanism with the buttress plate. The buttress plates remained loose and unscrewed to allow the coupling process to be assessed. The pelvis models were prepared for installation in a laparoscopic training torso, allowing for rapid interchangeability. We utilized standard laparoscopic grasping forceps along with a long screwdriver designed for pelvic surgery. Our optical tool was a standard 30-degree scope. The laparoscopic entry points were pre-configured on the trainer and could be selected as per standard laparoscopic procedures. The positioning of the trocar was determined in a manner that allowed for insertion of the 30-degree scope just above the navel in the epigastric region. The grasping forceps were inserted through a portal in the upper right quadrant, and the screwdriver was inserted through a portal in the lower left quadrant. In the model, the buttress plate could be manually inserted into the torso through the umbilical portal and transferred from the grasping forceps. After placing the plate within the torso, timing commenced as soon as it was no longer in the subject's hand. The subject's objective was to link the butress plate with its associated base plate, which had already been affixed. Successful completion of the connection, along with securing it in place using the screwdriver, was tallied as a successful attempt. Each subject underwent three such trials for every plate. If a trial extended beyond two minutes, it was halted, and a new one was initiated; such trials exceeding the time limit were recorded as unsuccessful. All subjects received standardized instructions and each trial began with standardized starting conditions. To eliminate any sequence-related bias, the subjects conducted the trials with the different plates in random order.

### Structure of the questionnaire

At the beginning, participants were required to provide details regarding their level of training and their prior experience with arthroscopy and laparoscopy. Following the completion of the three experiments involving a single plate, a questionnaire was administered. This questionnaire comprised the following five questions, with responses graded on a five-point Likert scale, aimed at assessing participants' perceptions of the handling of the various plates.

1st question: The insertion and transfer with the laparoscopic instruments of the modular plate into the model appears intuitive and smooth.

2nd question: The connection between plate and modular segment is complex and difficult to understand.

3rd question: The connection of the plate elements in the model is unproblematic and supported by the implant.

4th question: Disconnecting the plate elements in the model is unproblematic and supported by the implant.

5th question: The connection between the plate and the modular segment is stable.

### Statistical analysis

The hypothesis of this work is that there are differences in usability assessed on the basis of the time to reach the primary endpoint of successful implant placement. Trial time between plates was examined by ANOVA (with repeated measures). The post-hoc analysis was performed using a pairwise *T*-test with bonferoni correction. For the comparison of the continuous variable trial time by profession and actual endoscopic or arthroscopic experience, a *t*-test was applied.

The level of significance is set at 5% (*p* = 0.05). All analyses were completed using RStudio, version 1.2.5001 [Team R. RStudio: Integrated Development Environment for R; v. 1.2.5001. Boston, MA, USA: RStudio, Inc., 2019].

## Results

### Failed trials

**Figure 3:**
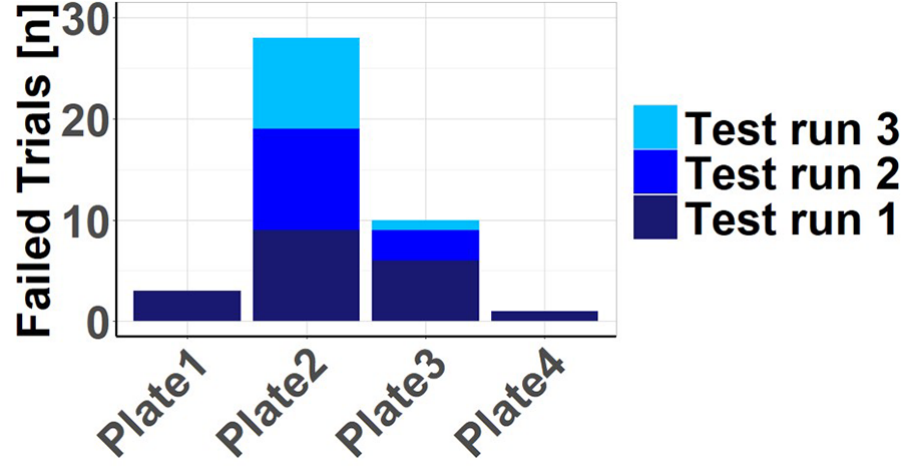
Failed trials: number of failed trials by plate and test run.

Failed trials are mainly found for connection design 2 [Total 27.45% (*n* = 28)]. Moreover, there was barely any enhancement observed with growing experience throughout the course of the test iterations [V1 26.47% (*n* = 9); V2 = 29.41 (*n* = 10); V3 26.47% (*n* = 9)]. Plate 3, while registering a considerable number of failed trials [Total 9.80% (*n*10)], also reflects a learning process, as the frequency of these failures diminishes over the course of the test runs [V1 17.65% (*n* = 6); V2 8.82 (*n* = 3); V3 2.94 (*n* = 1)]. In Plate 1 [Total 2.94% (*n* = 3)] and Plate 4 [Total 0.98% (*n* = 1)], instances of failure are rare and primarily occur during the first attempts, specifically among participants who are entirely new to the implant. This suggests that these two plates are characterized by their intuitive handling and demonstrate substantial learning improvements, particularly in contrast to Plate 2 (Figure 3).

### Time until implantation

**Figure 4:**
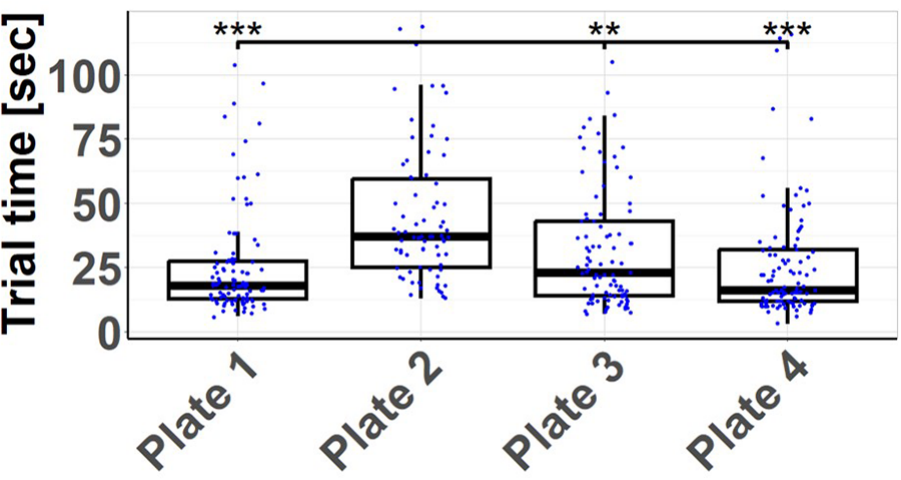
Trial times by plate: measured time to successful insertion of each plate. The handling time of plate 2 is significantly higher than that of the other plates. **p < 0.01. ***p < 0.001.

The trends observed in the analysis of failed attempts are consistent with the findings when examining the duration required for successful implantation. Plates 1 [25.6 ± 20.6 s] and 4 [25.7 ± 22.3 s] can be inserted most rapidly, whereas Plate 2 [44.9 ± 26.1 s] demonstrates the slowest performance. These differences were highly significant based on the ANOVA conducted [*p* < 0.001]. In post-hoc analysis, a highly significant [*p* < 0.001] discrepancy is evident when comparing Plate 2 with Plate 1 [*p* < 0.001; *d* = 0.74] and Plate 4 [*p* < 0.001; *d* = 0.59]. The speed at which Plate 3 [31.7 ± 23.1 s] could be managed and inserted during the tests falls in between. While there is a noticeable but not statistically significant variance in comparison to Plates 1 [*p* = 0.379] and 4 [*p* = 0.209], there is a significant difference compared to Plate 2 [*p* = 0.002; *d* = 0.45] (see Figure 4).

### Experience

**Figure 5:**
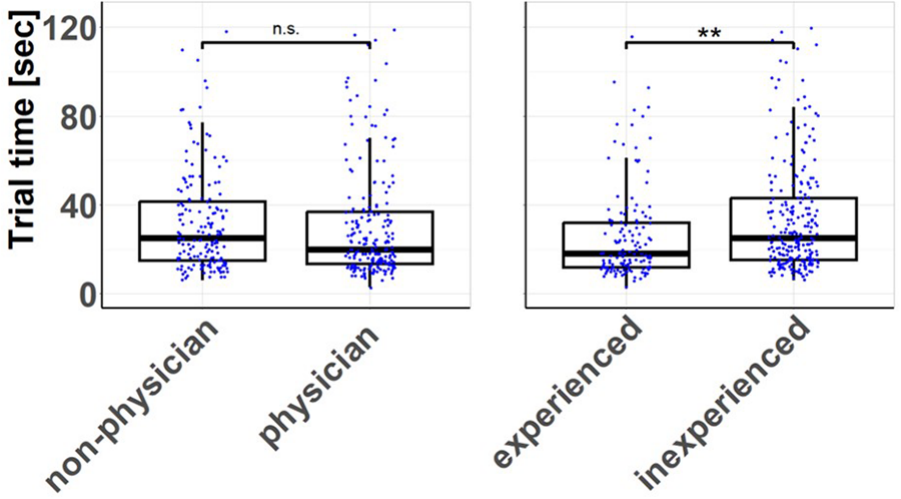
Trial times according to training and education level: measured time until successful insertion of the plates depending on profession and experience. There is no difference in handling times between physicians and non-physician participants. In contrast, participants with endoscopic or arthroscopic experience achieved a significantly reduced handling time compared to inexperienced participants. **p < 0.01.

Experience, while visually evident, does not significantly influence the speed at which individuals, whether physicians [30.4 ± 25.5 s] or non-physicians [32.0 ± 22.7 s], manage and insert the plate (*p* = 0.533). However, a different scenario emerges when we consider specific endoscopic or arthroscopic experience. Participants were considered experienced if they had a total of more than one year of experience in camera-assisted (arthroscopic or endoscopic) surgical techniques. Participants with such experience [26.2 ± 22.4 s] could significantly expedite the plate insertion process compared to their inexperienced counterparts [34.0 ± 25.0 s] (*p* = 0.002) (see Figure 5).

#### Learning curve

**Figure 6:**
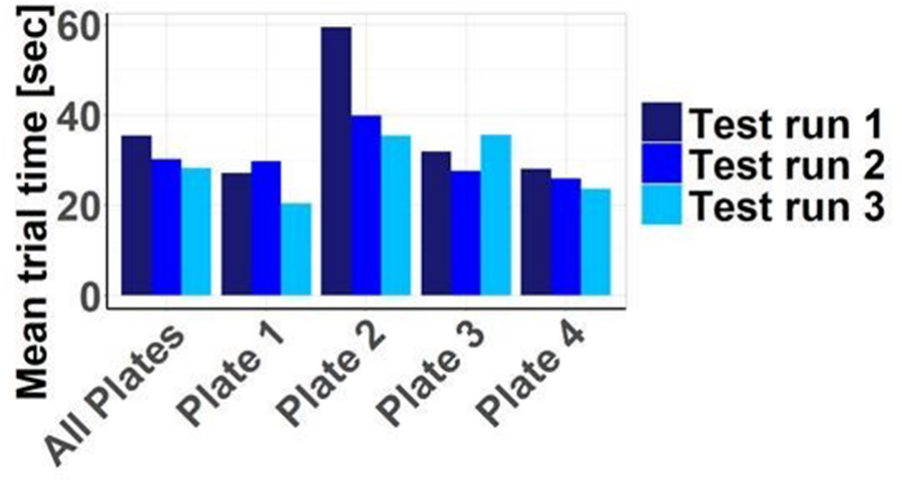
Trial times depending on plate and test run: overall, with increasing repetition, a learning success in the sense of reduced handling times becomes apparent. These are particularly prolonged for plate 2.

When examining the handling times segmented by plates and test runs, it provides context to the earlier suggestion of limited learning progress derived from the failed tests shown in Figure 3. In contrast, a notable learning curve becomes apparent regarding the time required, even though there were still failed attempts. Plates 1 and 3 exhibit in the 2nd and 3rd test runs deviations from a typical learning curve with consistently decreasing handling times, possibly due to the limited number of cases, which may be considered outliers. Plate 4, particularly when considering all plates together, demonstrates this learning curve effectively (see Figure 6).

#### Subjective evaluation

**Figure 7:**
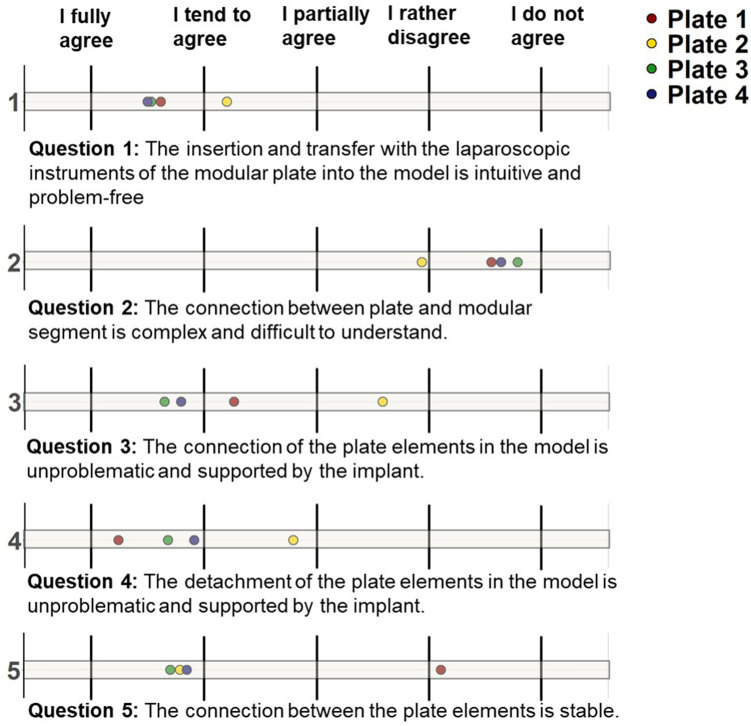
Subjective plate evaluation: evaluation of the handling of the plates based on 5 questions. Question 1: the insertion and transfer with the laparoscopic instruments of the modular plate into the model is intuitive and problem-free; Question 2: The connection between plate and modular segment is complex and difficult to understand; Question 3: the connection of the plate elements in the model is unproblematic and supported by the implant; Question 4: the detachment of the plate elements in the model is unproblematic and supported by the implant; Question 5: the connection between the plate elements is stable.

In addition to assessing objective handling characteristics based on timing and failed attempts, we also gathered the subjective impressions of participants through a set of 5 questions. The responses to the first question highlight that, in comparison to the other plates, the insertion and transfer of the 2nd plate, in particular, was less frequently perceived as intuitive and trouble-free. Correspondingly, the mechanical connection of the plate elements via the coupling mechanism is more often perceived as complex and challenging, especially for the 2nd plate according to question 2. For question 1 and question 2, the other plates received roughly similar assessments. Similarly, question 3 indicates that the mechanical connection of the plate elements via the coupling mechanism is less often seen as trouble-free for the 2nd plate. In plate 1, the mechanical coupling process is not perceived as straightforward and supportive by the implant, unlike in plates 3 and 4. This trend continues, with plate 2 being less often described as uncomplicated to detach from the manipulation instruments, while plate 1 is most frequently perceived as trouble-free in this regard. Plate 1 is also least often associated with a stable connection mechanism, whereas the connection mechanism for the other plates is generally perceived as stable. In summary, plates 3 and 4 receive the highest ratings in the subjective evaluation. Plate 1, on the other hand, presents more challenges in terms of connection, is easily detachable from the instruments, and has a perceived less stable coupling mechanism (see Figure 7).

## Discussion

In our current study, we investigated the practicality of different coupling mechanisms for a modular suprapectineal plate using a laparoscopic model. Our findings reveal that the grooved base plate (plate 1) and the pressure-sliding base plate (plate 4) outperform the sliding base plate (plate 2), exhibiting fewer failed attempts and significantly reduced trial times. Intriguingly, we observed that overall trial times do not significantly differ between non-physicians and physicians. However, participants experienced in endoscopic or arthroscopic procedures completed trials faster compared to their inexperienced counterparts. These results suggest that the grooved and pressure-sliding plates are not only intuitively manageable but also support effective learning.

Acetabular fractures pose a great surgical challenge due to the intricate acetabular anatomy and the heightened risk of iatrogenic nerve and vessel damage (8, 9). While elderly individuals often experience age-related pelvic fractures due to bone weakening, young adults between 20 and 30 years of age are more prone to “high-impact trauma,” often resulting from high-speed accidents or falls from significant heights (10, 11). These traumatic events subject the femoral head and acetabulum to substantial forces. While the “low-impact” fractures are often treated conservatively in the elderly population, the more severe pelvic fractures caused by large external forces, as well as dislocated pelvic fractures in the elderly, usually require surgical treatment to restore the functions of the pelvic ring or acetabulum (11).

Minimally invasive surgical procedures have been a major focus of clinical trauma research in the last decades. Regarding pelvic ring fractures, minimally invasive osteosynthetic procedures are already available and are widely used in clinical practice. These include the external fixator for injuries of the anterior pelvic ring (8, 11), or percutaneous sacroiliac (SI) screws for the posterior pelvic ring (12). For the acetabulum, less invasive anterior approaches such as the modified Stoppa or the pararectus approach have been developed (13, 14). In addition, specifically designed anatomic buttress plates allow for the stabilization of the quadrilateral surface or use of combined procedures with plate osteosynthesis of one column and additional lag screw osteosynthesis of the other column in acetabular fractures involving both columns, thus reducing the need for two-stage combined anterior and posterior surgery (2, 15). To further reduce the surgical trauma, endoscopic procedures have emerged for treating pelvic ring and acetabular fractures (2). In fact, David et al. (16) demonstrated recently that an extraperitoneal endoscopic dissection of the pelvis offers a similar visibility when compared to an open approach (16). Moreover, Hartel et al. (17) introduced in a cadaver study a fully endoscopic approach for anterior intrapelvic surgery and plate osteosynthesis (17). However, to ensure successful osteosynthesis, especially with such a minimally invasive approach, a feasible implant technology is required. Hence, we developed for the present study, a modular suprapectineal plate (7) with various coupling mechanisms and compared their performance to determine the most straightforward and intuitive handling method.

Our findings indicate that the grooved base plate (plate 1) and the pressure-sliding plate (plate 4) stand out with fewer failed attempts and significantly reduced trial times compared to the other plate designs. This advantage may be attributed to their ability to adjust their positioning after coupling. The grooved plate allows movement along its grooves, from ventral to dorsal, and thus, enabling different angles. Similarly, the pressure-sliding plate can be maneuvered in an oval window, flexibly adjusting the hole for module plate fixation within a 3 mm range, rendering it more practical. Conversely, the sliding base plate (plate 2) is associated with a longer test duration and a higher rate of failed trials, likely due to its less intuitive nature and complexity in threading at the correct angle, requiring simultaneous manipulation of both ends. Notably, endoscopic experience significantly influenced process time overall, underscoring the importance of regular endoscopic training when embarking on novel minimally invasive procedures, regardless of other clinical or surgical expertise. These results are in line with other clinical studies demonstrating that both training and experience in endoscopic procedures are of vital importance for achieving appropriate operative results. Findeklee et al. (18) demonstrated that interval training significantly improved the endoscopic knotting skills of medical students, regardless their acquired or individual factors (18). Moreover, Dyke et al. (19) showed that simulation training with a fixed curriculum significantly strengthened the performance in endoscopic surgery (19). In fact, endoscopic skills and their refinement seem to be independent of the overall surgical experience, as indicated by a recent study of Popa et al. (20) who developed a novel step-by-step training program for transanal endoscopic surgery (20). These findings are line with our results, which show no significant difference in the trial time of physicians and non-physicians, highlighting the importance of regular and monitored training in these minimal invasive methods.

One of the major strengths of our study is the inclusion of non-physicians and physicians, as well as endoscopic experienced and inexperienced personal, therefore providing a wide range of different skill and experience levels of the participants. Nonetheless, the present study also suffers from some limitations. These include that our experimental model only addressesd the evaluation of plate positioning. Successful surgery, however, also requires a careful tissue preparation without harming neurovascular structures and an adequate fracture reduction, which could not be simulated by our present model. Moreover, future studies must evaluate the mechanical stability of these implants by measuring bending stiffness or using finite element methods.

In summary, this study marks the initial exploration of various coupling mechanisms for a novel modular osteosynthesis plate using a laparoscopic model. Our results underscore the superior performance of coupling mechanisms featuring sliding grooves (plate 1) and a pressure-sliding design (plate 4) in terms of trial times, trial count, and subjective feasibility. Future research should extend this evaluation to cadaver studies, particularly in the realm of minimally invasive acetabular surgery in clinical practice.

## Data Availability

The raw data supporting the conclusions of this article will be made available by the authors, without undue reservation.
